# Treatment Responses in Histologic Versus Molecular Diagnoses of Lung Rejection

**DOI:** 10.3389/ti.2024.12847

**Published:** 2024-07-26

**Authors:** A. Zajacova, M. Mackova, K. Halloran, P. Gauthier, J. Balko, M. Guney, D. Rakita, M. Svorcova, J. Kolarik, J. Vachtenheim, J. Pozniak, J. Simonek, L. Fila, R. Lischke, P. F. Halloran, J. Havlin

**Affiliations:** ^1^ Prague Lung Transplant Program, Department of Pneumology, Second Faculty of Medicine, Charles University, Motol University Hospital, Prague, Czechia; ^2^ Alberta Transplant Applied Genomics Centre, Edmonton, AB, Canada; ^3^ Department of Medicine, University of Alberta, Edmonton, AB, Canada; ^4^ Department of Pathology and Molecular Medicine, Second Faculty of Medicine, Charles University and Motol University Hospital, Prague, Czechia; ^5^ Second Faculty of Medicine, Charles University, Prague, Czechia; ^6^ Prague Lung Transplant Program, 3rd Department of Surgery, First Faculty of Medicine, Charles University, Motol University Hospital, Prague, Czechia

**Keywords:** lung transplantation, acute cellular rejection, histopathology, gene expression, molecular biology

## Abstract

Histologic evaluation of allograft biopsies after lung transplantation has several limitations, suggesting that molecular assessment using tissue transcriptomics could improve biopsy interpretation. This single-center, retrospective cohort study evaluated discrepancies between the histology of transbronchial biopsies (TBBs) with no rejection (NR) and T-cell mediated rejection (TCMR) by molecular diagnosis. The accuracy of diagnosis was assessed based on response to treatment. 54 TBBs from Prague Lung Transplant Program obtained between December 2015 and January 2020 were included. Patients with acute cellular rejection (ACR) grade ≥ 1 by histology received anti-rejection treatment. Response to therapy was defined as an increase in FEV1 of ≥ 10% 4 weeks post-biopsy compared to the pre-biopsy value. Among the 54 analyzed TBBs, 25 (46%) were concordant with histology, while 29 (54%) showed discrepancies. ACR grade 0 was found in 12 TBBs (22%) and grade A1 ≥ 1 in 42 TBBs (78%). Treatment response was present in 14% in the NR group and in 50% in the TCMR group (*p* = 0.024). Our findings suggest that low-grade acute cellular rejection is less likely to be associated with molecular TCMR, which might better identify lung transplant recipients who benefit from therapy.

## Graphical Abstract

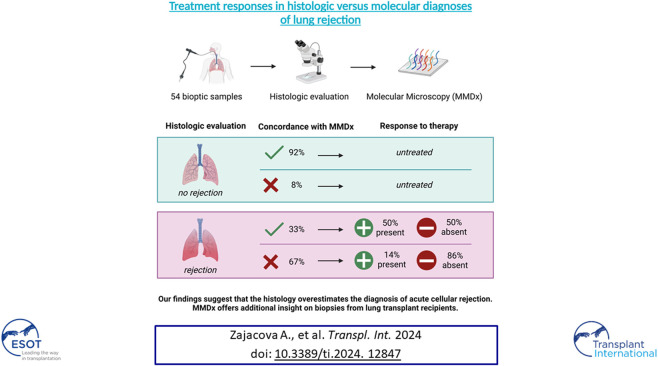

## Introduction

Lung transplant recipients (LTRs) face the shortest long-term survival among all of the major solid organ transplant recipients, with median survival of 6.7 years [1]. Lungs, as an open system in constant communication with the environment, possess an efficient immune complexity that serves a beneficial purpose as a barrier to infections. On the other hand, the potency of this system leads to a high rate of immune-mediated complications—of those, acute cellular rejection (ACR) is the most prevalent, affecting both morbidity and survival [2].

The management of ACR is limited by problems with the available diagnostic tools. The non-invasive tools routinely used for graft health monitoring, such as pulmonary function tests or radiological methods, lack both sensitivity and specificity for the diagnosis of ACR. Transbronchial biopsy (TBB) remains the gold standard for obtaining the diagnosis, despite its numerous limitations. Histologic evaluation is based solely on the abundance of perivascular and peribronchiolar lymphocytes, overlooking the composition and function of the immune cell subsets [3]. Obtained biopsy samples differ in size and quality and as demonstrated in the LARGO study, inter-pathologist interpretation of transbronchial biopsy for ACR is highly variable and limitedly reproducible [4].

Given the mentioned limitations, therapeutic strategies especially in minimal and mild ACR (grade 1 and 2) remain variable and often depend on the clinical condition of the patient, as well as on the preference of the physician [5].

Molecular analyses performed by the Molecular Microscope^®^ Diagnostic System (MMDx) may allow us to overcome the limitations of histopathology by performing microarray analysis of numerous transcripts, followed by both unsupervised and supervised analysis. This approach, already established as a standard-of-care in heart and kidney biopsies, aims to differentiate between diverse pathophysiological pathways of both immune- and injury-mediated processes, offering promising precision in distinguishing ACR from histopathologically similar conditions, such as regulatory or reperfusion changes in LTRs.

Despite the promising results of the INTERLUNG study, demonstrating lower variability and higher accuracy in assessing T-cell mediated rejection (TCMR) by MMDx in comparison to histology [6, 7], to the best of our knowledge no study to date has compared the accuracy of MMDx to standard histologic diagnosis in relation to treatment response. In this cohort, we aimed to describe the discrepancies between TBBs classified as no rejection (NR) and TCMR by MMDx compared to conventional histopathological evaluation and to assess the diagnostic accuracy of both methods based on patient treatment responses. Our assumption was that the more accurate diagnosis of TCMR would correspond to a greater response to TCMR treatment.

## Materials and Methods

### Study Population

We conducted a single-center retrospective cohort study based on prospectively collected transbronchial biopsies with all relevant clinical data obtained through in-depth review of the patients’ medical records.

**FIGURE 1:**
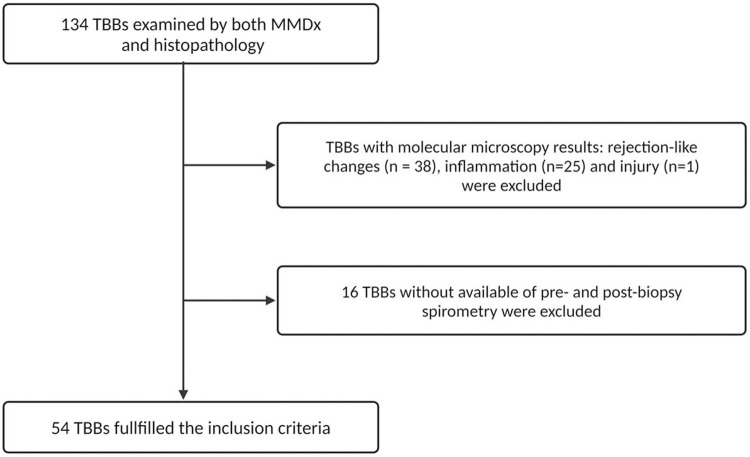
Consort diagram of the TBBs included in the study.

A total of 134 TBBs from patients transplanted between November 2004 and October 2018 were obtained between December 2015 and January 2020. All TBBs were examined by both histology and MMDx as a part of the multi-center INTERLUNG study (ClinicalTrials.gov #NCT02812290). For the purposes of our study, we selected TBBs that exhibited results of NR and definite TCMR according to MMDx with available pre- and 4 weeks post-biopsy spirometry. TBBs with rejection-like changes, inflammation, and injury detected by MMDx were excluded as our focus was on evaluating discrepancies in TCMR detection between MMDx and histology (Figure 1).

All of the included LTRs diagnosed with ACR grade A1 or higher by histology received anti-rejection treatment (corticosteroids, antithymocyte globulin, or alemtuzumab). Response to treatment was assessed 4 weeks after the biopsy and defined as an increase in percent predicted forced expiratory volume in 1 s (FEV1%) ≥ 10% of FEV1% before biopsy. To assess the decline in function at the time of TBB, we calculated the FEV1 decline as the ratio of the FEV1% expected (the mean of the last two FEV1% measurements prior to the pre-biopsy value) to the pre-biopsy FEV1%.

Surveillance biopsies were performed at 1st, 3rd, 6th and 12th month after lung transplantation (LuTx). Biopsies for cause were indicated based on the clinician’s decision, mainly due to a drop in lung function and/or radiological correlation. All patients with bioptically proven rejection received treatment. The center’s therapeutic protocol consists of corticosteroids in the first line, with oral escalation to 50 mg of prednisone and subsequent tapering for asymptomatic A1, and high-dose methylprednisolone (MP) for ACR grade ≥ A2 and symptomatic A1 rejection. Second-line treatment options, including anti thymocyte globulin (ATG) or alemtuzumab, were administered when initial corticosteroid therapy failed to demonstrate benefit. This study was approved by the Motol University Hospital Ethics Board. A written consent was obtained from each patient whose TBB specimens were used.

### Histology

TBBs for the histopathology evaluation were obtained during both surveillance and indication cryobiopsies. All histologic samples were fixed in neutral buffered 4% formaldehyde, postfixed and embedded in paraffin. The paraffin blocks were then sectioned into 4-µm-thick histological sections and stained with hematoxylin-eosin, Masson’s trichrome, orcein, Prussian-blue and periodic acid-Schiff staining.

All TBBs were also examined using immunohistochemistry for the following markers: CD45RO, CD8, CD4, CD20 and C4d. Three µm thick histologic sections were used, and each sample was stained using the following antibodies and protocols: anti-CD45RO antibody (mouse monoclonal antibody, clone UCHL1 [Agilent–Dako, Santa Clara, CA, United States], dilution 1:300, pre-treatment by heating in a buffer solution of pH6 in a water bath), anti-CD8 antibody (mouse monoclonal antibody, clone: C8/144B [Dako], dilution 1:200, pre-treatment by heating in a buffer solution of pH9 in a water bath), anti-CD4 antibody (mouse monoclonal antibody, clone: 4B12 [BioGenex], dilution 1:250, pre-treatment by heating in a buffer solution of pH9 in a water bath), anti-CD20 antibody (mouse monoclonal antibody, clone: L26 [Dako], dilution 1:300, pre-treatment by heating in a buffer solution of pH6 in a water bath) and anti-C4d antibody (mouse monoclonal antibody, clone ZM78 [ZETA Corporation, Sierra Madre, CA, United States], dilution 1:150, pre-treatment by heating in a buffer solution of pH9 in a water bath). The detection was performed using a one-step micro polymeric non-biotin system (Bio SB—Bioscience for the World, Santa Barbara, CA, United States) with a peroxidase and 3,3′-diaminobenzidine tetrahydrochloride solution. The nuclei were counterstained with hematoxylin. TBBs were evaluated by three LuTx-focused pathologists.

### MMDx

TBBs for the MMDx analysis were procured during a standard cryobiopsy procedure, during which two samples were collected. From the larger one, a small piece of tissue (2 mm × 2 mm × 2 mm) was excised and stored in RNA-later, which was followed by immediate freezing down to −70°C, while the rest of the tissue was used for a histopathological examination. The frozen biopsy specimens were then shipped in batches on dry ice to the Alberta Transplant Applied Genomics Centre/TSI (Edmonton, AB, Canada) for RNA extraction, labeling (3’ IVT plus labeling kit), and hybridization to PrimeView™ GeneChips^®^ (Applied Biosystems, Thermo Fisher Scientific, United States) by MMDx diagnostic system. The data were preprocessed using robust multiarray averaging. Genome-wide mRNA measurements were used to assign each biopsy a molecular diagnosis. An ensemble of supervised and unsupervised machine-learning algorithms trained on gene expression data from a large reference set of lung transplant biopsies, including expanded dataset of 744 TBBs (all surfactant level) with a subset of 600 TBBs (high surfactant level), was used to classify biopsies into four archetypal groups—No Rejection (NR), TCMR, rejection-like and inflammation [6, 7]. Only TBBs with NR (absence of inflammation/rejection transcripts) and definite TCMR (effector T-cell transcripts and INFG effects) were further included in the study, as depicted in the consort diagram (Figure 1). TBBs with rejection-like and inflammation archetypes identified by a higher expression of injury/repair, macrophage and endothelial-associated transcripts, but lacking T-cell associated transcripts, were excluded. This exclusion criterion was applied to maintain the study focus on assessing TCMR detection through MMDx.

### Statistical Analysis

All statistical analyses and visualizations were performed using GraphPad Prism 9.0 (San Diego, United States) and R version 4.1.3.^1^ Wilcoxon-Mann-Whitney and Fisher’s exact tests were used for continuous and categorical variables, respectively. The correlation between FEV1 decline and response to treatment (FEV1% change 4 weeks after biopsy) was evaluated using Spearman’s correlation. Both univariate and multivariate logistic regression analyses were conducted to identify significant predictors of treatment response. Odds ratios (OR) with 95% confidence intervals (CI) and *p*-values were calculated to determine the statistical significance of each predictor. A *p*-value of less than 0.05 was considered statistically significant.

## Results

### Patient and Biopsy Characteristics

In this study, 134 TBBs were analyzed by both MMDx and histopathology. The MMDx results identified 17 cases of definite TCMR (12.7%), 53 NR (39.6%), 38 rejection-like changes (28.3%), 25 inflammation (18.7%) and 1 injury (0.8%). TBBs exhibiting rejection-like changes, inflammation and injury archetypes were excluded from further analysis as well as 2 TBBs with TCMR (11.8%) and 14 with NR (26.4%) due to the unavailability of either pre-biopsy spirometry or 4-week follow-up spirometry. 54 TBBs obtained from 41 LTRs fulfilled the inclusion criteria: 15 with TCMR (27.8%) and 39 with NR (72.2%) identified by MMDx. Histological examination revealed ACR grade A0 in 12 (22.2%), grade A1 in 24 (44.4%), grade A2 in 15 (27.8%), and grade A3 in 3 TBBs (5.6%).

Among the included patients, the median age at LuTx was 54.0 years (IQR 33.0–60.6), 18 were female (43.9%). The pre-biopsy spirometry was conducted on average 3 days prior to biopsy (SD 5.3 days) and the 4-week follow-up spirometry on average 29.9 days post-biopsy (SD 9.4 days). 36 of the LTRs underwent bilateral LuTx (87.8%), while 5 patients (12.2%) underwent unilateral transplantation. The primary diagnosis included COPD in 17 (41.5%), IPF in 11 (26.8%), ILD in 4 (9.8%) and CF in 9 (21.9%) patients (Table 1). The median time between LuTx and biopsy was 13.4 months (IQR 4.4–39.5). 21 TBBs were performed for surveillance purposes (38.9%) and 33 were indicated for a cause (61.1%). All LTRs who underwent surveillance TBB were asymptomatic at the time of biopsy. Among those biopsied for cause, a significant difference was observed: 90% of ACR/TCMR patients were symptomatic at the time of TBB, in contrast to 39% of ACR/NR patients (*p* = 0.016; see Table 2).

**TABLE 1:** Patient characteristics: TBBs with histological ACR grade ≥ A1 (ACR+) were divided into two groups, based on the presence of T-cell mediated rejection (TCMR) and no rejection (NR) by MMDx.

	ACR+/TCMR (n = 14)	ACR+/NR (n = 28)	*p*-value
Age at LuTx (years; median, IQR)	27.3 (20–57)	51.1 (21.3–59.5)	0.13
From LuTx to TBB (days; median, IQR)	517 (345–1,251)	486 (128–1,596)	0.44
Female (n, %)	7 (50)	15 (54)	0.99
Primary diagnosis			0.79
Chronic obstructive pulmonary disease (n, %)	4 (29)	9 (32)	
Interstitial lung disease (n, %)	1 (7)	1 (4)	
Idiopathic pulmonary fibrosis (n, %)	2 (14)	7 (25)	
Cystic fibrosis (n, %)	7 (50)	11 (39)	
Pulmonary hypertension (n, %)	0	0	
Other (n, %)	0	0	
LuTx type			0.28
Double (n, %)	14 (100)	24 (86)	
Single (n, %)	0	4 (14)	
Infection at TBB			0.35
Viral (n, %)	1 (7)	0	
Bacterial (n, %)	2 (14)	5 (18)	
Mycological (n, %)	0	0	
None (n, %)	11 (79)	23 (82)	
DSA at TBB			0.23
Class I (n, %)	0	1 (4)	
Class II (n, %)	3 (21)	1 (4)	
None (n, %)	10 (71)	20 (71)	
Not performed (n, %)	1 (7)	6 (21)	
High-dose corticosteroids within 3 months prior to TBB (n, %)	2 (14)	5 (18)	0.99
Chronic lung allograft dysfunction at TBB			0.19
Bronchiolitis obliterans syndrome (n, %)	5 (36)	6 (21)	
Restrictive allograft syndrome (n, %)	1 (7)	0	
Mixed (n, %)	0	0	
Undefined (n, %)	0	0	
None (n, %)	8 (57)	22 (79)	

**TABLE 2:** Biopsy-related characteristics: TBBs with histological ACR grade ≥ A1 (ACR+) were divided into two groups, based on the presence of T-cell mediated rejection (TCMR) and no rejection (NR) by MMDx.

	ACR+/TCMR (n = 14)	ACR+/NR (n = 28)	*p*-value
From LuTx to TBB (days; median, IQR)	517 (345–1,251)	486 (128–1,596)	0.44
Reason for TBB			0.73
Surveillance (n, %)	4 (29)	10 (36)	1
Symptomatic (n, %)	0	0	
Asymptomatic (n, %)	4 (100)	10 (100)	
Indication (n, %)	10 (71)	18 (64)	0.05
Symptomatic (n, %)	9 (90)	9 (50)	
Asymptomatic (n, %)	1 (10)	9 (50)	
ACR grades			
A grade			0.02
Grade 1 (n, %)	5 (36)	19 (68)	
Grade 2 (n, %)	6 (43)	9 (32)	
Grade 3 (n, %)	3 (21)	0	
Grade 4 (n, %)	0	0	
B grade			0.25
Grade 0 (n, %)	1 (7)	6 (21)	
Grade IR (n, %)	3 (21)	9 (32)	
Grade IIR (n, %)	1 (7)	0	
Grade X (n, %)	9 (64)	13 (46)	
C grade			0.19
Grade 0 (n, %)	3 (21)	13 (46)	
Grade 1 (n, %)	2 (14)	1 (4)	
Grade X (n, %)	9 (64)	14 (50)	
Treatment			
Surveillance			1
Corticosteroids (n, %)	4 (100)	10 (100)	
Indication			0.71
Corticosteroids (n, %)	7 (70)	15 (83)	
Anti-thymocyte globulin (n, %)	2 (20)	2 (11)	
Alemtuzumab (n, %)	1 (10)	1 (6)	
Response to treatment	7 (50)	4 (14)	0.03
Surveillance (n, %)	0	0	
FEV1% decline at TBB (median, IQR)	9.3 (−2.2–14)	3.7 (0.4–8.5)	0.61
FEV1% before TBB (median, IQR)	52.7 (51.9–60.6)	94.9 (66.8–108.1)	0.04
FEV1% 4w after TBB (median, IQR)	56.1 (43.5–73.3)	91.4 (67.5–112.3)	0.05
FEV1% change (%)	0.8 (−18.5–6.6)	−0.5 [(−9.5)–6.6]	0.95
Indication (n, %)	7 (70)	4 (22)	0.02
FEV1% decline at TBB (median, IQR)	−22.1 [−30.8–(−15.8)]	−11.3 [−14.5–(−7.8)]	0.01
FEV1% before TBB (median, IQR)	37.6 (27.9–62.5)	65.5 (47.5–72.9)	0.03
FEV1% 4w after TBB (median, IQR)	54.0 (33.5–73.3)	62.9 (50.1–75.8)	0.41
FEV 1% change (median, IQR)	15.7 (−2.1–37.5)	2.1 (−6.5–7.9)	0.07

### Agreement Between Histology Diagnosis and MMDx

**FIGURE 2:**
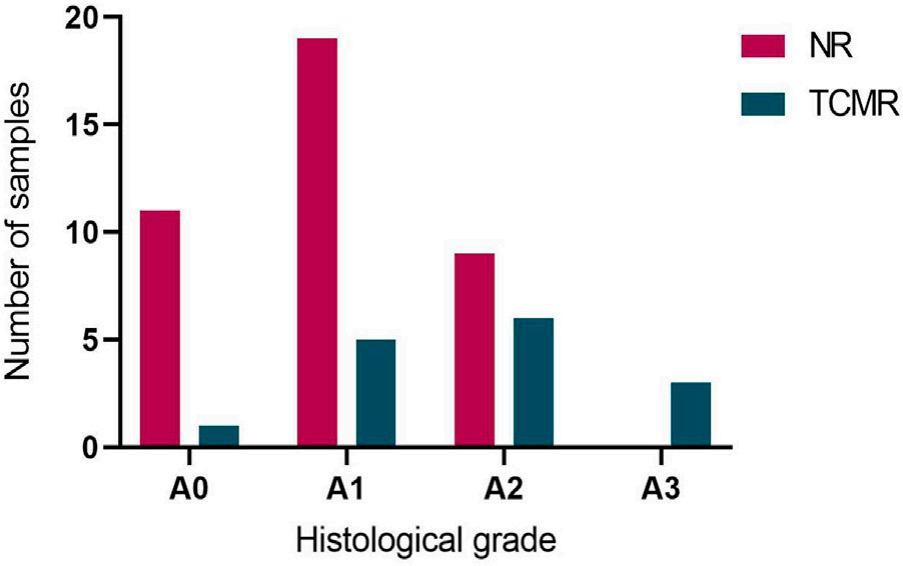
Agreement between histological acute cellular rejection (ACR) grade A and diagnosis of no rejection (NR) and T-cell mediated rejection (TCMR) by MMDx. NR by MMDx was considered concordant with ACR grade A0 and TCMR concordant with ACR A grade ≥ A1.

**FIGURE 3:**
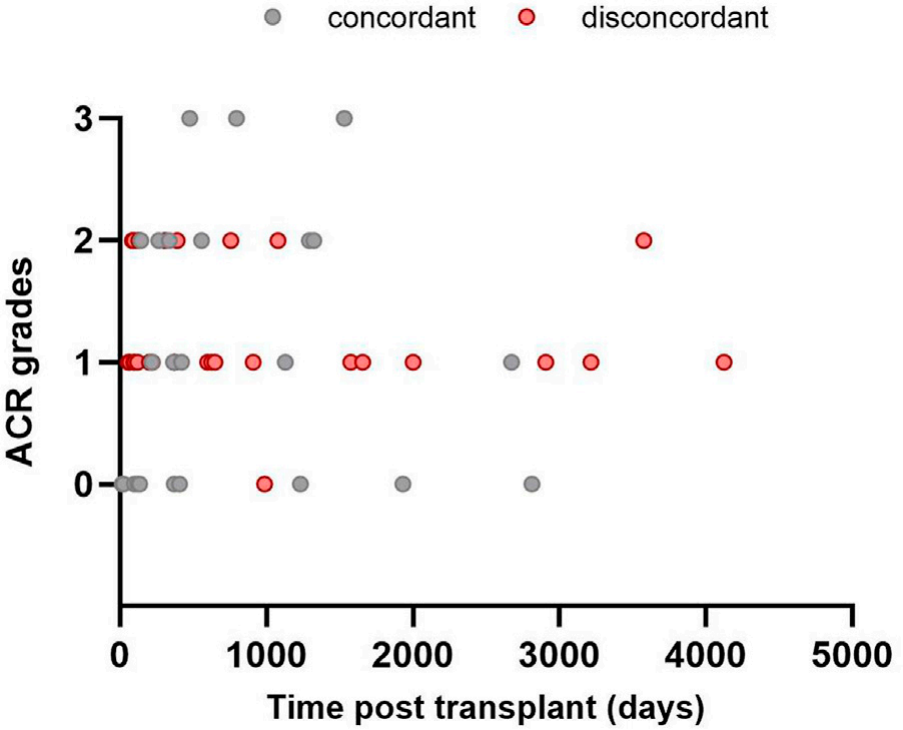
Time distribution of included transbronchial biopsies: agreement between histology (ACR; grades A0–A3) and MMDx. No rejection (NR) by MMDx was considered concordant with ACR grade A0 and T-cell mediated rejection (TCMR) concordant with ACR A grade ≥ A1.

Overall, the MMDx was concordant with the histologic diagnosis in 25 TBBs (46.3%), while discrepancies were observed in 29 (53.7%) of them. Among TBBs with ACR grade A0, agreement with the MMDx was present in 11 (91.7%; ACR-/NR) and only one biopsy showed a discrepancy (8.3%; ACR-/TCMR). Of the 42 patients with ACR ≥ A1 by histology, TCMR (ACR+/TCMR) was present in 14 of them (33.3%). For ACR grade A1, consistency was found in only 5 TBBs (20.8%; A1/TCMR), while discordance between histology and MMDx was observed in 19 TBBs (79.2%; A1/NR). Among the 15 TBBs with ACR grade A2, matching results were present in 6 (40%; A2/TCMR) and absent in 9 (60%) cases (A2/NR). All of the TBBs with ACR grade 3 agreed with the TCMR diagnosis by MMDx (A3/TCMR; Figures 2, 3).

### Response to Treatment

All patients with ACR ≥ A1 by histology (n = 42) received treatment—36 with corticosteroids (85.7%), 4 with ATG (9.5%) and 2 with alemtuzumab (4.8%). A response to treatment was observed in 11 LTRs (29.9%): 10 with corticosteroids (90.9%) and 1 with alemtuzumab (9.1%). 31 LTRs were unresponsive (73.8%).

Patients with ACR+/TCMR (n = 14) received therapy: 9 with MP pulses (64.3%), 2 with prednisone escalation (14.3%), 2 with ATG (14.3%), and 1 with alemtuzumab (7.1%). Seven patients (50%) responded to the treatment: 6 with MP pulses (85.7%) and 1 with alemtuzumab (14.3%).

In the ACR+/NR group, 19 patients received MP pulses (67.9%), 6 prednisone escalation (21.4%), 2 ATG (7.1%), and 1 alemtuzumab (3.6%), of whom 4 LTRs treated by MP pulses (14.3%) demonstrated a response. We found a significant difference when comparing the molecular NR and TCMR groups: 4 LTRs (21.1%) in the molecular NR group (ACR+/NR) and 7 LTRs (50.0%) in the molecular TCMR group (ACR+/TCMR) responded to the treatment (*p* = 0.024).

Among the LTRs with ACR grade 1, 14 received MP pulses (58.3%), 8 prednisone escalation (33.3%), and 2 received ATG (8.3%). Only 4 (16.7%) responded to treatment, all treated with MP pulses, while 20 (83.3%) did not respond. Of the responders, 1 (20%) was from the TCMR group and 3 (15.8%) were from the NR group by MMDx. No significant difference in treatment response was found between A1/TCMR and A1/NR (Figure 5).

In the ACR grade 2 group, 14 LTRs (93.3%) received MP pulses and one (6.7%) received alemtuzumab. Six (40%) responded to the therapy: 5 (83.3%) from the TCMR group and 1 (11.1%) from the NR group by MMDx. There was a significant difference in treatment response between A2/TCMR and A2/NR (*p* = 0.01; see Figure 5).

Of the three patients with ACR grade A3, two received ATG without response (66.7%), and one received alemtuzumab with a positive response (33.3%). Both non-responders had CLAD at the time of biopsy. The first one was a patient with CLAD, grade 4 at time of biopsy, with persistent DSAs and received multiple anti-rejection therapies prior to included biopsy. The second one was a patient with a clinical diagnosis of steroid resistant ACR.

FEV1 decline is considered one of the major predictors of treatment response. Using Spearman correlation analysis, we found only a weak correlation between FEV1 decline and response to treatment, defined as FEV1% change 4 weeks after biopsy (r = 0.2, *p* = 0.21).

To evaluate TCMR as a predictor of treatment response, we performed logistic regression analyses adjusted for major potential confounders such as ACR grade and FEV1% decline at biopsy. Given the limited number of patients with ACR grade A3 (n = 3), for logistic regression analyses we combined ACR grades into two groups: ACR grade A1 (minimal) and ACR grades A2 + A3 (mild-to-moderate). This resulted in 24 patients in ACR grade A1 (57%) and 18 in ACR grades A2 + A3 (43%).

In the univariate logistic regression, both TCMR (*p* = 0.018) and FEV1% decline (*p* = 0.018) were significant predictors of treatment response, while the ACR grade was not (*p* = 0.11). In the multivariate analysis, none of the predictors reached statistical significance (Table 3). These results suggest that while TCMR and FEV1% decline are significant in univariate analysis, their effects are attenuated in the multivariate model, likely due to multicollinearity and the limited sample size.

**TABLE 3:** Univariate and multivariate logistic regression depicting predictors of treatment response—diagnosis of T-cell mediated rejection (TCMR), FEV1 decline at biopsy (ratio of the FEV1% expected to the pre-biopsy FEV1%), and grade of acute cellular rejection (ACR) with odds ratios (OR) with 95% confidence intervals (CI) and *p*-values determining the statistical significance of each predictor.

Univariate logistic regression		
Predictor	Odds ratio (95% CI)	*p*-value
TCMR	6.00 (1.41–29.23)	0.018
FEV1 decline	1.08 (1.02–1.17)	0.018
ACR grade	2.27 (0.84–6.64)	0.11

Multivariate logistic regression		
Predictor	Odds ratio (95% CI)	*p*-value
TCMR	2.96 (0.47–17.75)	0.23
FEV1 decline	1.07 (1.00–1.15)	0.07
ACR grade	1.69 (0.49–5.86)	0.39

To account for the expected increase in lung function within the first post transplant year, we compared the univariate logistic regression results for two different post-transplant periods. In the early period (0–365 days; n = 16, 38.1%), the effect of the FEV1 decline on treatment response had an OR of 1.23 (95% CI: 1.04–1.62, *p* = 0.055). In the late period (>365 days; n = 26, 61.9%), the OR was 1.08 (95% CI: 1.01–1.20, *p* = 0.065). We did not find a significant difference in the effect of the FEV1 decline on treatment response between the early and late post-transplant periods.

**FIGURE 4:**
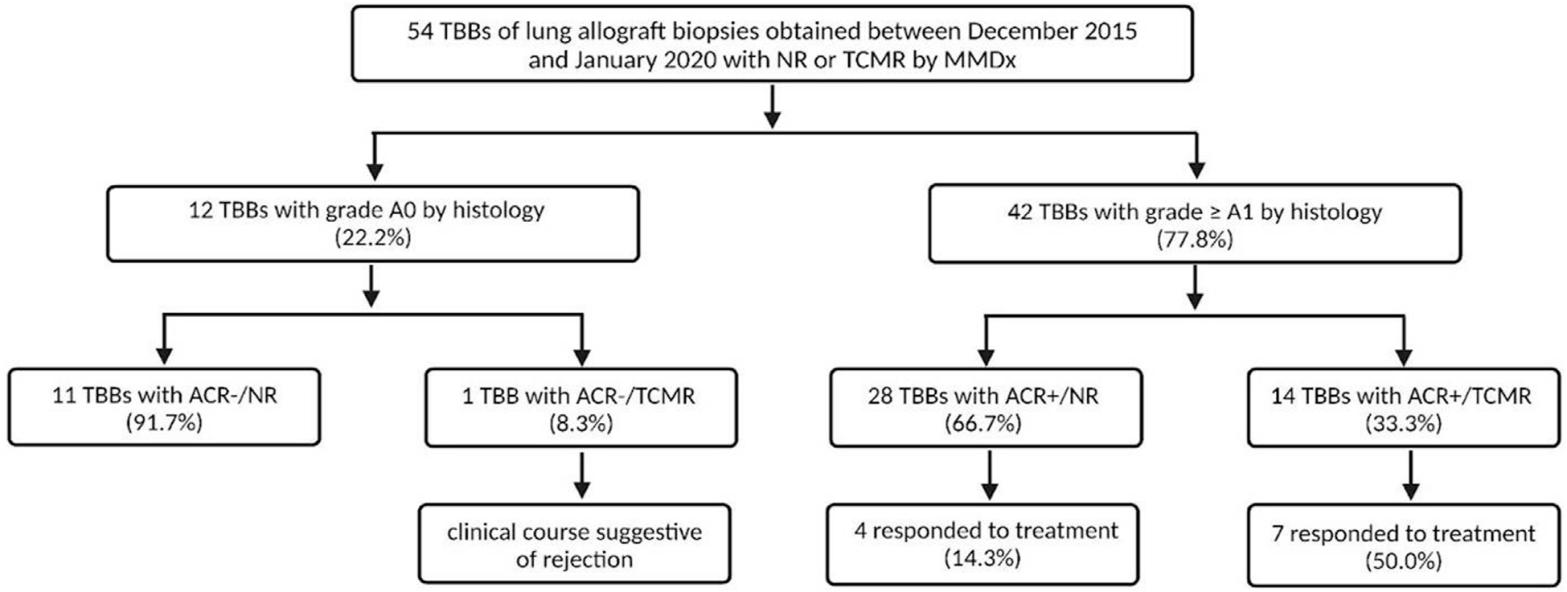
Differences in responses to anti-rejection treatment of acute cellular rejection (ACR) in TBBs with no rejection (NR) and T-cell mediated rejection (TCMR) by MMDx for both ACR A0 and grade ≥ A1 groups. Response to treatment was defined as an increase in FEV1% ≥ 10% of FEV1% prior to biopsy.

**FIGURE 5:**
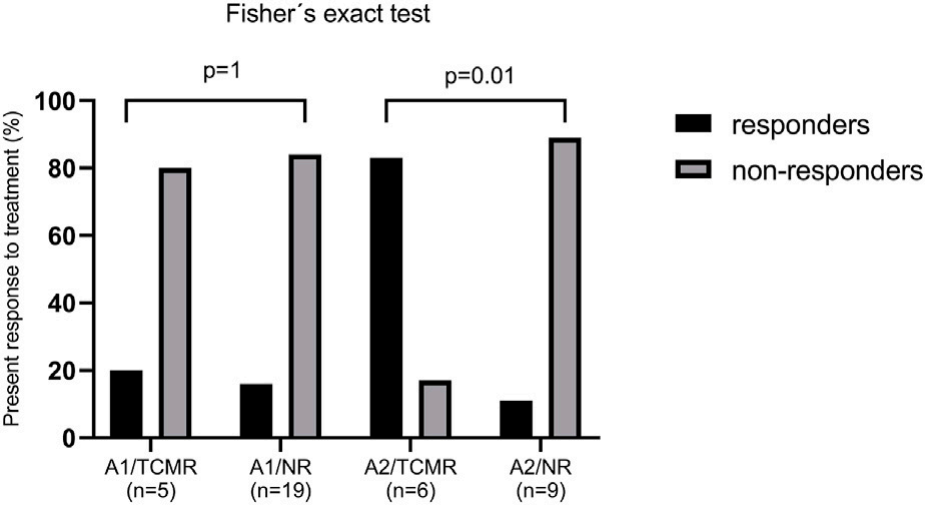
Anti-rejection treatment response in patients with histological acute cellular rejection (ACR) grade A1 and A2 in patients with no rejection (NR) and T-cell mediated rejection (TCMR) by MMDx. Response to treatment was defined as an increase in FEV1% ≥ 10% of FEV1% prior to biopsy.

Regarding the presence of DSAs at the time of TBB, three patients (21.4%) in the ACR/TCMR group and two patients (7%) in the ACR/NR group had DSAs. Notably, none of these patients responded to therapy. For further details, see Table 2 and Figures 3–5.

## Discussion

There is a need to develop novel, more sensitive biomarkers of graft alteration to guide patient management, given the limited survival of LTRs in comparison to other solid organ transplant recipients. Although data on some promising novel biomarkers for non-invasive monitoring have been published, such as donor-derived cell-free DNA (dd-cfDNA), Torque teno viral load, or exosomes, all of them lack specificity in identifying the underlying pathophysiological processes that lead to organ damage, failing to detect ACR specifically [8]. A molecular analysis of the tissue, despite its invasive nature, might offer a more specific understanding of the underlying graft pathology, providing a clear proof of TCMR, based on the presence of specific rejection-associated transcripts [6, 7]. MMDx has already been approved for use in clinical routine for kidney and heart transplant recipients [9, 10], but the MMDx approach in LTRs lacks more profound clinical data despite some very promising results in the INTERLUNG collaboration [6, 7, 11, 12]. In this retrospective study, we aimed to determine the accuracy of NR and TCMR diagnosis by MMDx in both surveillance and acute transbronchial biopsies based on treatment response and compare the results of this novel approach to the standard histological evaluation.

The absence of perivascular and peribronchial lymphocytic infiltrates (histological grade A0) should provide clear information, equal to a healthy allograft, as per current diagnostic criteria [13]. Nevertheless, recent publications in kidney transplant demonstrated disagreements between no rejection in histology and rejection by MMDx, ranging around 20% for antibody-mediated rejection and 40% for TCMR [14]. The factors possibly affecting the accuracy of the diagnosis include inter-pathologist disagreement and sampling variations [15]. Although the latter might affect both histology and MMDx [16], when combined with dd-cfDNA levels, MMDx correlates with survival in kidney transplants better than histology [17, 18]. Another additional value of a molecular approach was depicted in the publication by Schachtner et al. [19] in kidney transplantation, demonstrating superiority of MMDx in TCMR borderline lesions that have not yet met histological criteria for ACR. These findings suggest the potential superiority of MMDx over traditional histological evaluation for guiding clinical decisions.

Our study demonstrated a very good concordance between the mentioned methods in non-rejecting biopsies. There was only one biopsy with a histological finding of no rejection and TCMR by MMDx diagnosis (ACR-/TCMR). The further clinical course of this patient was suggestive of rejection, highlighting the fact that ACR, as well as other immune- and infection-mediated pathways in the lung, might often present with heterogeneous, patchy distributions, that might not be fully represented in the bioptic sample. While a histological evaluation in these limited biopsies may not fulfill the diagnostic criteria for ACR, the presence of specific transcripts in the tissue could be detected by MMDx.

High consistency was also observed in moderate ACR (grade A3) TBBs by histology—MMDx demonstrated TCMR in all three of these biopsies. Thus, in our cohort, MMDx showed a strong concordance in lung biopsies with either absent or moderate rejection by histological assessment, but when it comes to minimal and mild rejection (grade A1 and A2, respectively), the results vary significantly between histology and MMDx, with an overall discordance rate of 72% (79% for ACR grade A1 and 60% for grade A2). We hypothesized that the presence of lymphocytic infiltrates, particularly in lower grades of rejection, might not necessarily indicate rejection, but could instead signify other pathological processes, especially if MMDx does not concurrently reveal the presence of TCMR-specific transcripts. In order to assess the accuracy of the diagnosis in the discrepant results, the presence of response to treatment was taken into consideration. In the ACR+/NR group, only 14% of patients presented with an improvement of lung function following an anti-rejection therapy, compared to 50% in the ACR+/TCMR group. These findings support the superiority of MMDx evaluations in a context of clinical decision-making. However, when analyzed for A1 and A2 grades separately, statistically significant difference in response to therapy was observed only in A2 samples (Figure 5). Moreover, none of the included LTRs treated with peroral prednisone escalation (treatment of choice for A1 grade in this cohort) showed response to treatment. Supporting this, Levy et al. demonstrated that the first untreated grade A1 rejection in spirometrically stable recipients within the first posttransplant year was not significantly associated with a risk for CLAD or death [3]. Especially in the early postoperative period, there is a wide variety of processes apart from rejection going on, including postischemic and reperfusion damage, as well as infectious complications. The absence of TCMR in biopsies showing minimal or mild ACR might be explained by the fact that the cell subpopulations and other immune components of similar-appearing ACR lesions may differ significantly between patients and may have different correlations with lung injury, even though the ISHLT criteria for ACR were met. The molecular approach provided by MMDx might overcome these limitations, however, its utility in our limited retrospective cohort was not demonstrated for samples with minimal rejection.

Within the ACR+/TCMR group, half of the LTRs (n = 7; 50%) responded to the therapy. Notably, 43% of the non-responders in ACR ≥ A1 by histology and TCMR by MMDx (ACR+/TCMR) had chronic lung allograft dysfunction (CLAD) present at the time of biopsy and received multiple courses of high-dose corticosteroids prior to biopsy. This observation raises the question of whether the failure to improve after corticoid therapy was in fact due to established CLAD changes. It is possible that patients with ongoing CLAD and TCMR might benefit from a more aggressive therapeutic approach. Further studies are required regarding this topic.

For lung transplant recipients, the incorporation of MMDx into the diagnostic routine might provide additional insights into the graft pathology, especially addressing the clinically challenging asymptomatic lower-grade acute cellular rejection, similarly to borderline findings in kidney transplants. Its utility might further clarify the necessity of treatment, especially in surveillance biopsies in the absence of other signs of rejection, preventing the unnecessary use of intensive anti-rejection protocols, and minimizing the risk of their significant adverse effects such as increased susceptibility to infections.

Our study has certain limitations, as it is a single-center retrospective study with a modest size of the cohort and the exclusion of TBBs with inflammation, rejection-like changes, and injury from the analysis. These archetypes, if incorporated into future studies on larger patient cohorts, might aid in clarifying the discrepancy of results in the case of a histological diagnosis of ACR with concurrent absence of TCMR-specific transcripts identified by MMDx.

In summary, our findings suggest that low-grade acute cellular rejection is less likely to be associated with molecular TCMR, which might better identify patients who benefit from therapy and offer additional insights into biopsies of lung allografts. While further research is required, our promising pilot data suggest that MMDx has the potential to become a routinely used tool for diagnosing TCMR in lung transplant patients.

## Data Availability

The raw data supporting the conclusions of this article will be made available by the authors, without undue reservation.

## Footnotes

1
www.r-project.org

## Abbreviations

ACR, acute cellular rejection; ATG, antithymocyte globulin; CF, cystic fibrosis; CLAD, chronic lung allograft dysfunction; COPD, chronic obstructive pulmonary disease; dd-cf DNA, donor-derived cell-free DNA; FEV1%, percent predicted forced expiratory volume in 1 second; ILD, interstitial lung disease; IPF, idiopathic pulmonary fibrosis; LuTx, lung transplantation; LTRs, lung transplant recipients; MMDx, Molecular Microscope Diagnostic System; NR, no rejection; TBB, transbronchial biopsy; TCMR, T-cell mediated rejection.
